# Responding to health inequities: Indigenous health system innovations

**DOI:** 10.1017/gheg.2016.12

**Published:** 2016-08-22

**Authors:** J. G. Lavoie, D. Kornelsen, L. Wylie, J. Mignone, J. Dwyer, Y. Boyer, A. Boulton, K. O'Donnell

**Affiliations:** 1 Community Health Sciences, University of Manitoba College of Medicine, Winnipeg, Manitoba, Canada; 2 Western Centre for Public Health and Family Medicine, University of Western Ontario Schulich School of Medicine and Dentistry, London, Ontario, Canada; 3 Health Care Management, Flinders University, Adelaide, South Australia, Australia; 4 Social Sciences, Brandon University, Brandon, Manitoba, Canada; 5 Health and Development, Whakauae Research for Māori Health and Development, Whanganui, New Zealand

**Keywords:** Aboriginal, primary healthcare, primary care, equity, self-determination

## Abstract

Over the past decades, Indigenous communities around the world have become more vocal and mobilized to address the health inequities they experience. Many Indigenous communities we work with in Canada, Australia, Latin America, the USA, New Zealand and to a lesser extent Scandinavia have developed their own culturally-informed services, focusing on the needs of their own community members. This paper discusses Indigenous healthcare innovations from an international perspective, and showcases Indigenous health system innovations that emerged in Canada (the First Nation Health Authority) and Colombia (Anas Wayúu). These case studies serve as examples of Indigenous-led innovations that might serve as models to other communities. The analysis we present suggests that when opportunities arise, Indigenous communities can and will mobilize to develop Indigenous-led primary healthcare services that are well managed and effective at addressing health inequities. Sustainable funding and supportive policy frameworks that are harmonized across international, national and local levels are required for these organizations to achieve their full potential. In conclusion, this paper demonstrates the value of supporting Indigenous health system innovations.

## Introduction

In 2006, The Lancet published a series of papers focused on Indigenous health [1–4], calling for international action to address Indigenous health inequities. This series reported that world-wide, Indigenous peoples remain easily displaced, are generally undervalued and in some cases are disappearing altogether as a result of systemic exclusion and policy neglect, or through more active measures such as violent interventions. Health inequities and differential treatment are related to the history of Indigenous–settler interactions, a misguided and failed ideology of tutelage, competition over resources located on Indigenous lands, power imbalances, and cultural differences [5]. Recent decades have seen a resurgence of Indigenous-led activism, culminating with the 2007 United Nations Declaration on the Rights of Indigenous Peoples (UNDRIP). UNDRIP was initially adopted by 144 countries world-wide, with later support from Australia, Canada, Colombia, New Zealand, Samoa and the USA.

In many countries (e.g., Australia, Canada, the USA [6]), Indigenous peoples have drawn on the discourses of Indigenous rights and health equity to highlight the failures of mainstream services, including health services, in meeting their needs, while advocating for policies supporting community-managed health services. In other countries (Colombia, New Zealand), free market ideologies have created some institutional space for Indigenous communities to enter the healthcare market as service providers [7, 8]. In Scandinavian countries, Norway being a salient example, national discourses of equality have hampered opportunities for the creation of more responsive Indigenous-led services operating in parallel to mainstream services [9].

Our objective in this paper is to discuss Indigenous healthcare innovations from an international perspective, highlighting key constitutional, legal and organizational characteristics. We draw upon case studies of Indigenous health system innovations that have emerged in Canada (the First Nation Health Authority) and Colombia (Anas Wayúu), as examples of Indigenous-led innovations to serve as models to other communities.

## The international context

Worldwide, colonial histories, current demographic and national socio-economic status are some of the many factors that shape Indigenous-state relations, and create or limit access to responsive care. Table 1 provides an overview of selected characteristics for each country, comparing Indigenous rights and jurisdictions, as well as funding, accessibility and cultural appropriateness of health care services.

**Table 1.** Cross-national comparisons *[*6, 7, 9, 24, 30–36*]*

	Aotearoa (NZ)	Australia	Canada	Colombia	Norway	The USA
Indigenous pop. (as % of total pop)	600 000 (15%)	669 000 (3%)	1.4 M (4%)	1.4 M (4%)	137 000 (3%)	2.9–5 M (1.1%)
Jurisdiction for Indigenous Affairs	New Zealand Government	Split between State/Territory and Commonwealth governments. Commonwealth took up active role in 1973	Federal since Confederation (1867) First Nation since Confederation, Inuit since 1939 – In Re: Eskimo and Metis 2016, Daniels	No specific jurisdiction	No specific jurisdiction	Federal government since 1954
Indigenous Rights	Based on the 1840 Treaty of Waitangi	Since the 1992 Mabo case, land title pre-existing the conquest are recognized. The special relationship between the Commonwealth government remain largely policy-based. Constitutional recognition is under discussion	Based on the Royal Proclamation, 1763, and in the Treaties. Reaffirmed in section 35 of the Constitution [1982 (Aboriginal and Treaty rights)]. The Indian Act (1985) however limits the sphere of influence of these documents, and benefits are tied to on-reserve residence. Bolstered by federal commitments to UNDRIP	1991 Constitutional reform extended Indigenous rights to political autonomy, cultural protection and territorial integrity	Article 110a of the Constitution (1988) states: ‘It is the responsibility of the authorities of the State to create the conditions enabling the Sami people to preserve and develop its language, culture and way of life’	Constitutional provisions limited to commerce. Tribal nations are characterized under U.S. law as ‘domestic dependent nations’, which is understood as a guarantee of sovereignty
Jurisdiction for Indigenous Health	Department of Health since 1911	Split between State/Territory and Commonwealth government since 1973	Federal, with Health Canada since 1944	Constitutional commitment to full coverage for healthcare, since 1991	No specific jurisdiction	Federal, Indian Health Services since 1955
Health care system	Tax financed primary, secondary and tertiary care with access fee for primary health care and a private care counterpart. Exemption for the poor, but they must register to qualify	Tax financed primary, secondary and tertiary care. Public hospital and some PHC treatments are free. Co-payments apply to medicines and many medical and diagnostic services. Exemption for the poor, but they must register to qualify	Tax financed primary, secondary and tertiary care with no access fee	A Contribution Regime (CR), which covers workers and their families with monthly incomes above a minimum monthly amount, and the Subsidized Regime (SR) covers those identified as poor. CR is financed by mandatory payroll tax contributions and national and local tax revenues. SR comes from taxation	Tax financed primary, secondary and tertiary care with no access fee	Tax financed primary, secondary and tertiary care with access fee and a private care counterpart. Indian Health Services
Funding for Indigenous controlled health services	Funding comes through the same mechanisms as other providers such as District Health Boards, although other funding comes from the Māori Health Directorate, as a result of Treaty responsibilities	Services emerged in 1971 from community mobilization, and short term project funding from both Commonwealth and state governments followed. More stable core funding plus project funding since 1995, but more fragmented and less stable than funding for mainstream PHC	Core funding based on historical expenditures plus three percent indexation, capped for the population existing at the time of signature. Project funding and new initiatives generally introduced on competitive basis	Central government funds Empresas Promotoras de Salud (EPS)	N/A	American Indian Self-Determination and Education Assistance Act (Public Law 638 adopted in 1975)
Main limitations on culturally appropriate services	Fragmented funding; competition; underfunding and lack of support in some District Health Boards	Fragmented funding; competition; underfunding and lack of legislative and infrastructure support	Underfunding and defunding, jurisdictional fragmentation between prevention, primary, secondary and tertiary care undermining continuity of care	Underfunding, political instability, lack of state commitment	An ideology of equality that makes parallel services unappealing to central government	Underfunding and defunding, jurisdictional fragmentation between prevention, primary, secondary and tertiary care undermining continuity of care

### Constitutional recognition

Constitutional recognition varies across countries. Both Canada and the USA recognize some measure of Indigenous nationhood. The US Constitution recognizes Indigenous nations as ‘domestic dependent nations’ although whether or to what degree this entails a formal recognition of tribal sovereignty vis-à-vis the USA or individual states remains a topic of debate [10, 11]. Canada's Constitution includes explicit protections for ‘Aboriginal and Treaty rights’ as well as an affirmation of a ‘nation-to-nation’ relationship between Canada (or the Crown) and Indigenous nations, which has remained intact since first contact with European powers [see the Constitution Act 1982: Ss25, 35, 12]. These measures are also defined to some degree in local and provincial policies as well as modern treaties and self-government agreements. New Zealand does not have a written constitution: Māori have continuously argued that the Treaty of Waitangi, signed in 1840, serves as their Constitution, and also guarantees a measure of sovereignty to iwi (tribes). Both Colombia and Norway have made recent constitutional amendments, recognizing the right to cultural autonomy. Colombia also recognizes a right to political autonomy (arguably a form of sovereignty [7]) whereas Norway does not [13]. Although there is no formal recognition of Indigenous sovereignty within Australia's Constitution, there is a statutory recognition of ‘Aboriginal title’ to traditional lands that survived the unilateral extension of British sovereignty through colonization [see 14]. Again, the extent to which this entails rights to legal and political jurisdiction over said lands, remains a topic of considerable debate [15, 16]. Currently, discussions are under way regarding the formal constitutional recognition of Indigenous peoples as the original inhabitants.

### Access to responsive health services

In all countries under study, health inequities remain, and are often linked to systemic exclusion, discrimination and racism. Around the world many Indigenous communities have responded with the development of Indigenous controlled health services. In Canada and the USA, legislation (USA only) and policy (Canada) created opportunities for nations or tribes to take over the delivery of health services previously managed and delivered by federal health services (the Indian Health services in the USA, and the First Nations and Inuit Health Branch in Canada), in the name of sovereignty or self-government, starting at the level of the First Nations band or tribe, moving into larger collaborative arrangements across a group of communities, to the recent initiative in British Columbia (BC) that created a transfer of previously funded and in some cases managed health services to a First Nations organization that represents all 203 bands in the province.

In New Zealand and Colombia, new Indigenous-controlled health services emerged through Indigenous health organizations competing for health services delivery contracts. In New Zealand, this opportunity emerged as a result of a shift towards privatization of health services that fragmented health services previously developed by government-managed health boards into a multiplicity of contracts Māori and other health organizations could compete for. In Colombia, opportunities emerged as a result of a 1991 Constitutional commitment to full coverage for healthcare. In Australia, Indigenous controlled health services emerged as a result of community mobilization, in response to unmet needs and racism. The 1995 establishment of the Commonwealth Office for Aboriginal and Torres Strait Islander Health resulted in increased funding for a network of Indigenous controlled health services, and increased access to responsive primary health care (PHC) for Aboriginal peoples. In Norway, the Sámi community never advocated for separate health services, except in the area of mental health. The *Sámisk nasjonalt kompetansesen*ter (SANKS, created in 2002) provides low threshold mental health services for Sámi, with funding from Helse Nord (the Health Authority). SANKS emerged as a result of Sámi advocacy. And while SANKS is likely to continue because of needs, there remains little support for Sámi-centric services in Norway [9].

### International covenants

Over the past four decades, International Covenants have multiplied, raising the global profile of inherent Indigenous rights (see Table 2). International covenants are not binding documents, and must be incorporated into domestic law to have enforceability: they may be seen as aspirational, not prescriptive. They nevertheless raise the profile of Indigenous rights, and provide a lever for domestic discussion. In Canada, for example, the federal government's recent public commitment to the UNDRIP principles has led at least one province to legislate a statutory commitment to the principles of the UNDRIP [17] as well as a federal effort to harmonize Canadian laws with the UNDRIP [18]. The UNDRIP, to which all countries included in this study are now signatories, is regarded as equivalent to established principles of international law. It is also an important document for advancing inherent rights for Indigenous peoples.

**Table 2.** International covenants, conferences and their relevance to indigenous health

		Countries that are signatories of the covenant (indicated as “yes”)					
Covenant	Relevance	Aotearoa (NZ)	Australia	Canada	Colombia	Norway	The USA
International Covenant on Civil and Political Rights (CCPR) United Nations 1966 [37]	1: right to self-determination for all peoples (not specifying indigenous peoples), right to freedom of movement (12), of religion and belief (18), of opinion (19) and of assembly (21) constrained by the need to protect public health 27: right for minorities to practice their culture, profess and practise their own religion, or use their own language Establishes the authority of the UN Human Rights Committee to hear grievances, ratified by Can, OZ & NZ	Yes	Yes	Yes	Yes	Yes	Yes
ILO Convention No. 169 Concerning Indigenous and Tribal Peoples in Independent Countries 1989 [38]	7.2 2. The improvement of the conditions of life and work and levels of health and education of the peoples concerned, with their participation and co-operation, shall be a matter of priority in plans for the overall economic development of areas they inhabit. Special projects for development of the areas in question shall also be so designed as to promote such improvement.	No	No	No	Yes, 1991	Yes	No
	20.2. Governments shall do everything possible to prevent any discrimination between workers belonging to the peoples concerned and other workers, in particular as regards:						
	(c) medical and social assistance, occupational safety and health, all social security benefits and any other occupationally related benefits, and housing;						
	24. Social security schemes shall be extended progressively to cover the peoples concerned, and applied without discrimination against them.						
	25. 1. Governments shall ensure that adequate health services are made available to the peoples concerned, or shall provide them with resources to allow them to design and deliver such services under their own responsibility and control, so that they may enjoy the highest attainable standard of physical and mental health.						
	25.2. Health services shall, to the extent possible, be community-based. These services shall be planned and administered in co-operation with the peoples concerned and take into account their economic, geographic, social and cultural conditions as well as their traditional preventive care, healing practices and medicines.						
	25.3. The health care system shall give preference to the training and employment of local community health workers, and focus on primary health care while maintaining strong links with other levels of health care services.						
	25.4. The provision of such health services shall be co-ordinated with other social, economic and cultural measures in the country						
United Nations Declaration on the Rights of Indigenous Peoples 2007 [39]	*Article 21,* 1. Indigenous peoples have the right, without discrimination, to the improvement of their economic and social conditions, including, inter alia, in the areas of education, employment, vocational training and retraining, housing, sanitation, health and social security.	Yes, 2009	Yes, 2009	Yes, 2010	Yes, 2009	Yes, 2007	Yes, 2010
	2. States shall take effective measures and, where appropriate, special measures to ensure continuing improvement of their economic and social conditions. Particular attention shall be paid to the rights and special needs of indigenous elders, women, youth, children and persons with disabilities.						
	*Article 23* Indigenous peoples have the right to determine and develop priorities and strategies for exercising their right to development. In particular, indigenous peoples have the right to be actively involved in developing and determining health, housing and other economic and social programmes affecting them and, as far as possible, to administer such programmes through their own institutions.						
	*Article 29*, 1. Indigenous peoples have the right to the conservation and protection of the environment and the productive capacity of their lands or territories and resources. States shall establish and implement assistance programmes for indigenous peoples for such conservation and protection, without discrimination.						
	2. States shall take effective measures to ensure that no storage or disposal of hazardous materials shall take place in the lands or territories of indigenous peoples without their free, prior and informed consent.						
	3. States shall also take effective measures to ensure, as needed, that programmes for monitoring, maintaining and restoring the health of indigenous peoples, as developed and implemented by the peoples affected by such materials, are duly implemented						

## Looking closer: Anas Wayúu and the First Nations Health Authority of BC

### Anas Wayúu in Colombia†^1^

In Colombia, the 1991 Constitutional reform recognized the inherent autonomy for Indigenous groups to exercise the right to their own legislative and judicial powers within their territories. Other reforms included a commitment to guarantee full healthcare coverage. In 1993 the Colombian government passed Law 100, creating Health Promoting Enterprises (*Empresas Promotoras de Salud–EPS*). The EPSs are financed through two different systems. For segments of the population that can afford it, there is a contributory regimen. The other subsidized system seeks to provide coverage to the population with less ability to pay, and is funded in part by input from the contributory system and by government subsidies.

The Wayúu people, one of the approximately 80 Indigenous ethnic groups in Colombia, live in La Guajira, Colombia next to the Caribbean (as well as in the northeast region of Venezuela). The Wayúu population in Colombia is estimated to be 380 000, representing 24% of the Indigenous population in the country, and 45% of the population in La Guajira [19]. The majority live in small rural villages and hamlets spread across the region. The dominant housing style is a wood frame plastered with mud, while buildings constructed of concrete blocks and cement are less common. Most communities lack running water in the houses and have no electricity, although some households have generators. Access to clean water is a concern in most Wayúu communities [20]. Similar to other rural Indigenous areas in Colombia, the completion of education is limited, with no more than 33% of children that start school completing grade 12. Approximately 18% of those living in rural communities are illiterate or functionally illiterate [21].

Pilot government information systems initiatives on population health suggest that the Wayúu's epidemiological profile is linked to poverty, with some specific aspects related to geography (scarcity of water) and culture (social organization and economic activity). The most frequent pathologies include: malnutrition, respiratory and gastrointestinal infections among children under 5 years of age, sexually transmitted infections, uterine/cervical cancer, hypertension, injuries due to interpersonal violence, caries and other dental problems among all ages [22].

The Indigenous EPS Anas Wayúu was created in 2001 by two Indigenous associations representing 120 Indigenous communities: the *Association of Cabildos and/or Traditional Authorities* of la Guajira, and the *Sumuywajat Association*. The administration of Anas Wayúu is accountable to these associations in terms of its direction. Anas Wayúu has an enrollment of 118 000 people. It is responsible for providing coverage for primary, secondary and tertiary healthcare services, as well as health promotion programs. Most of the employees of Anas Wayúu are bilingual in Wayúunaiki and Spanish. Anas Wayúu offers the services of bilingual guides for Wayúu families who do not speak Spanish or who prefer to communicate in Wayúunaiki [19].

As a not-for-profit health insurance company, Anas Wayúu provides healthcare coverage through a wide network that includes small health centres, clinics and hospitals in La Guajira, as well as cities in other regions of Colombia. Services include preventive and health promotion programs, out-patient consultations with physicians and dentists, basic surgery, laboratory work, basic radiology, and the provision of essential drugs. For urgent care and hospitalization, Anas Wayúu contracts services across the country (although mostly in La Guajira). The services include emergencies, in-patient hospital care, surgeries, childbirth, and care of the newborn. It also contracts with two high complexity health institutions, for Cancer, HIV/AIDS, renal insufficiency, severe burns, cardiac care, and intensive care. Anas Wayúu also supports community programs linked to traditional Indigenous medicine practices, seeking to collaborate with and complement the Western health system. Intercultural and holistic health and care are central notions of Anas Wayúu's mission [23], resulting in responsive care and improved outcomes [24].

### The First Nations Health Authority in Canada^2^

Although Canada`s First Nations peoples are a matter of federal constitutional jurisdiction, they access the vast majority of their health services from provincial Departments of Health, including access to mainstream hospitals, family physicians and specialists. Few if any of these services are delivered on First Nations reserves. The federal government has historically, and continues currently, to fund and in some cases deliver a limited complement of services focused on prevention, home care and in some remote communities, primary care delivered by nurses with an expanded scope of practice. Despite recent legal debates that articulate the fiduciary obligations of the federal government around Indigenous health in Canada, the federal government continues to assert that services are provided as a matter of policy only for humanitarian reasons and not due to any Aboriginal or Treaty rights [25].

For decades, this dual funding system (federal – provincial) has generated debates as to who is responsible for expenditures, resulting in confusion, frustration, delays, increased morbidity and premature mortality [26–28], not only related to health funding, but also in areas that impact the determinants of health, such as housing and education. Part of the issue is related to federal program authorities and accountability frameworks, which have over the past decade become more tightly targeted in their definition of program eligibility. This shift is linked to budget cuts, increased scrutiny over public expenditures, and concerns that discretion might result in preferential treatment of some over others [28]. Recent trends have been for a literal and conservative interpretation of policies, causing delays and denials [26].

To date the only province equipped to effectively address this issue is BC. The 2011 *Framework Agreement for First Nations Health Governance in BC* between the Government of Canada, the Government of BC and the First Nations Health Society initiated a new model of health governance for First Nations in BC, including strategies for increasing First Nations control over health care services delivery throughout the province [29]. This agreement, which began to be discussed in 2005 following the demise of the Kelowna Accord,^3^ set the stage for the creation of the First Nations Health Authority (FNHA), which took over the responsibility for the funding and development of on-reserve services from BC region of the First Nations Inuit Health Branch of Health Canada in October 2013. Although most on-reserve health programs are run by First Nations bands through contracts with the federal government, the FNHA took over those contracts to support band run programs. The First Nations Health governance structure evolved to include the First Nations Health Council (leadership and advocacy), the First Nations Health Directors Association (advisory and professional development) and the FNHA (service delivery).

The FNHA is working with the province and the Regional Health Authorities (funded by the provincial government) to address the gaps in health services through increased coordination and collaboration across mainstream health services and the First Nations communities it serves. Through this process of jurisdictional transfer, BC First Nations developed regional tables to support the improved cooperation and coordination between the five Regional Health Authorities and First Nations representatives, to identify the priorities of the First Nations communities in the region, as well as the responsibilities of the Regional Health Authorities to ensure that First Nations needs are met and that people are treated with respect in the health system. The innovative and aspirational goals not only aim to improve accessibility and cultural safety for First Nations in the mainstream health services, there are efforts underway to refocus health services away from a sickness model to one that incorporates a holistic perspective of wellness based on First Nations values of a balance between physical, mental, emotional and spiritual health [40].

### Opportunities and challenges in operationalizing these models

Both case studies report on models that emerged because of a policy-enabling opportunity. Anas Wayúu emerged to answer unmet service delivery needs, 8 years after the adoption of Law 100, which opened opportunities for the creation of EPSs. The First Nations Health Authority emerged also as a result of unmet needs, in a federal policy context favoring smaller governments, increased provincial engagement in addressing the healthcare needs of First Nations, and coincidentally Indigenous self-government. As such, both initiatives presented answers to key policy problems. The creation of Anas Wayúu nevertheless required the creation of a health delivery infrastructure, whereas the First Nations Health Authority was a transfer of existing federal structures and programs, which nevertheless require considerable transformation.

To date, both innovations have been closely scrutinized. Despite reporting good relationships with government officials, and being awarded the status of best EPS in Colombia, Anas Wayúu has nevertheless noted close scrutiny at different stages of its development. Likewise, the First Nations Health Authority has experienced national media scrutiny reflecting both (and at time simultaneous) enthusiasm and some skepticism given the scale of the project, which is unprecedented anywhere in the world.

It is clear that both organizations are expected to produce improved health outcomes despite serving communities where continued economic and social marginalization is the norm, under heightened scrutiny, and while being tasked of transforming mainstream institutions and practice. This is by all accounts a tall order.

At an operational level, both innovations occupy an uncomfortable space positioned simultaneously within a self-determination and Indigenous rights paradigm, and a commissioning health services paradigm, with defined contractual obligations and performance indicators. As discussed elsewhere, these two paradigms are not easily reconciled [30].

## Discussion and conclusion

Increased international attention to Indigenous rights, which are encoded in international covenants, may well be legitimizing pathways that Indigenous communities are already forging, in the pursuit of autonomy and better health. This convergence is important to note. When supported by international, national, and local policy frameworks, Indigenous health organizations are able to address health system and organizational lacunae, and provide coordinated and culturally appropriate care. It is therefore important that local and national governments not only work to harmonize their legislation and policy frameworks with existing international and constitutional parameters, but that they do so in an inclusive manner that is informed by Indigenous expertise.

In addition, it is critical to note that Indigenous peoples will continue to use mainstream health services, particularly for specialized care that is out of the scope of Indigenous run services. Therefore, it is crucial to ensure that these services are culturally safe and informed of the preferences and issues local Indigenous populations face. This must be an ongoing priority, alongside increased Indigenous control of health services in their communities.

Further, in order for Indigenous controlled health services to succeed in improving health inequities, governments must ensure that policy frameworks move towards harmonization with norms regarding Indigenous autonomy, and that they are bolstered with adequate funding to enable Indigenous communities to succeed in their pursuit of the right to health and well-being. With this support, Indigenous innovations stand to address health inequities by transforming services under their purview, but also health services provided to Indigenous peoples by mainstream services. This is essential to addressing continued health inequities, and to implement the spirit of international covenants, Treaty obligations (where they exist) and Indigenous rights.

## Footnotes

† The notes appear after the main text.

## Notes

1. See web site at http://epsianaswayuu.com/.

2. See web site at http://www.fnha.ca/.

3. The Kelowna Accord was series of agreements between the Government of Canada, First Ministers of the Provinces, Territorial Leaders, and the leaders of five national Indigenous organizations in Canada. The Accord sought to improve the education, employment, and living conditions for Indigenous peoples through governmental funding and other programs. Despite extensive discussion, the Accord was never endorsed by the Government of Stephen Harper.
